# Supplementary material for: Chronic wounds in Sierra Leone: Searching for Buruli ulcer, a NTD caused by Mycobacterium ulcerans, at Masanga Hospital
Source: PLoS Negl Trop Dis. 2021 Oct 13;15(10):e0009862. doi: 10.1371/journal.pntd.0009862 (PMC8544828; doi:10.1371/journal.pntd.0009862)
Supplement: Attachment — Submitted filename: Letter of response re Revised Manuscript Buruli NIHR.docx [file pntd.0009862.s002.docx]

# **Letter of response**

Dear PLOS,

Thank you very much for returning a review of our manuscript "Chronic wounds in Sierra Leone: searching for Buruli ulcer, a NTD caused by Mycobacterium ulcerans." for consideration at PLOS Neglected Tropical Diseases.

We are very grateful for all the comments from experts in the field. In light of the reviews we have attached the resubmission of a significantly-revised version which we feel takes into account all your reviewers' comments.

We have attached the manuscript with tracked changes, as well as a clean version. Below are our responses to the review comments (in italics in order from corresponding email) with a description of the changes made in the manuscript, with reference to the lines where possible.

**Responses to review comments with descriptions of changes made in the manuscript.**

*Reviewer #2:*

*This is an observational crosssectional study, performed on a subset of a peoples with chronic wound received in a single study site, namely the Masanga Hospital. Therefore, even if the study design and the method are well described, I'll suggest, given the method of recruitment of participants, and the very low number of study participant, to reconsider the title of the manuscript as followed: Chronic wounds in Sierra Leone: searching for Buruli ulcer, a NTD caused by Mycobacterium ulcerans, at the Masanga Hospital.*

Response: We agree that this study is limited to a single study site on a subset of people with chronic wounds. We also agree that the title needs amending.

Change(s): Titles amended as suggested (Lines 3-5); these identified limitations are conferred in the Discussion section (Lines 927-31)

*Reviewer #1:*

*No. Results presentation is unclear

Reviewer #2:*

*The result section is well written*

Response: The reviewers had differing opinions on how the results section was presented. We therefore amended the results section based on specific comments from reviewers, described in the following responses.

*Reviewer #2:*

*The conclusion cannot be generalized to Sierra Leone, but only to the Masanga Hospital. Once the Title will be reconsidered, the conclusion will also be fine*

Response: Addressed above

*Reviewer #1:
Line 118: The authors state that surgical intervention is often required in BU. This is erroneous and needs to be corrected. Indeed, most cases of BU heal with only recommended combination antibiotic therapy (now oral clarithromycin and rifampicin) and appropriate wound dressing. At the present time, surgery is mainly adjunctive and may include procedures such as wound debridement and skin grafting. The treatment of BU including the role of surgery needs to be properly contextualised and discussed to avoid confusing non-experts who might read the manuscript.*

Response: We agree the wording was confusing and misleading regarding the surgical treatments sometimes required in BU treatment. We wanted to make the point that wounds with unknown aetiology often require surgical treatment, whereas BU is responsive to anti-mycobacterium therapy.

Change(s): Introduction updated to reflect the treatment of BU with antibiotics and wound dressings, whilst surgery has a role in wounds of unknown aetiology (Lines 135-144)

*Reviewer #1:*

*The case definition for BU used is not clear. The authors state that ‘Suspicion of BU was based on several criteria, including: a lesion exhibiting one of the classical four forms of BU (painless itchy nodules, plaques, small ulcerations, extensive ulcerations)…*

Response: We agree that the methods section needed re-wording to clarify which wounds were included.

Change(s): We have re-worded the methods section to make the inclusion criteria clearer, and added the comment that ‘Although both ulcerating and non-ulcerating lesions were included in the study design, no patients with non-ulcerating lesions suspicious of BU were identified in the study period.’ Previously we had not included this clarification as this statement is part of the results rather than the methods, however the reviewers comments suggest it is confusing to state in the methods that ulcerating and non-ulcerating wounds were included when only ulcerating wound are included in the results. The reason for this discrepancy was that non-ulcerating wounds were part of the inclusion criteria, however no such patients presented to the Hospital during the study period. (Lines 205-215)

We have also highlighted this issue as a limitation in the discussion session.

(Lines 933-39)

*Reviewer #1:*

*Were non ulcers e.g. nodules and plaques included in the study?*

*If only ulcers were included, why do the authors say that fine needle aspirates were collected (line 138-139)? FNA is only recommended for non-ulcerative lesions. The authors need to explain this. Further from line 213-216, the authors indicate no FNAs were taken as no patients with non-ulcerative lesions presented. This section and the previous one referred to supra makes reading confusing and difficult to understand. The authors should rationalise the 2 sections to improve readability and understanding*

Response: We agree that the methods section needed re-wording to clarify whether FNA was used.

Change(s): We have re-worded the methods and results section regarding FNAs to clarify that as both ulcerating and non-ulcerating wounds were in the inclusion criteria, the plan to carry out FNA to acquire tissue samples from any non-ulcerative lesions, however none such patients presented to Masanga during the study period and therefore this method of tissue sampling was not used. (Lines 269-72, 311-12, 405-410)

We have also highlighted this issue as a limitation in the discussion session.

(Lines 933-39).

*Reviewer #1:*

*Macrovascular complications of diabetes mellitus presenting with foot ulcers are an important cause of chronic lower limb ulcers in the West African sub-region. Did the authors not consider this aetiology? I find it difficult to appreciate why this important differential was not considered. Rather than focus on previous history of malaria and yellow fever, it will be more important to know which patients had history of diabetes and how diabetes mellitus was ruled out in others.*

Response: Diabetes mellitus was considered as an aetiology of the wounds, and was part of our exclusion criteria. As part of the screening and data collection, all patients were asked about history of diabetes mellitus, and no patients included reported of diabetes mellitus in their current or past medical history. Any patients at the hospital suspected as potentially having diabetes would have glucose testing routinely as per local hospital protocol, with none of the included patients having abnormal results. There are no medical records to verify any previous history, so this is reliant on patients self-reporting. Malaria and yellow fever were not a focus of the current and past medical history questions, it’s just these were the most frequently reported.

Change(s):

- Clarified diabetes mellitus explicitly as an exclusion criteria (Lines 207-215)
- Included further information in Methods on how and what past medical history was explored (Lines 227-232).
- Expanded on the co-morbidity findings, specifically in relation to diabetes, in the Results section (Lines 364-67).
- Clarified information in Table 2 with a note related to an asterisk ** in column 2 which underneath lists all the specific medical problems the patient was asked if they suffered with currently or previously, in order to display the information that no patients reported a diagnosis of diabetes (Page 17)
- Inclusion of the limitation of the history of diabetes and self-reporting nature of some of the information in the Discussion (Lines 819-24, 936-42)

*Reviewer #1:*

*While it is important to ascertain whether BU is present in Sierra Leone, it is not very helpful when the defined population for the study is not representative. In West Africa, BU typically occurs in persons <15 years and ulcers have been present for a relatively short duration. Why was BU being looked for in patients majority of whose ulcers had been present for longer than 1 year? Again, the age range of the population under study does not represent the typical BU population in West Africa. This is an important consideration as the likelihood of finding BU was low apriori*

Response: The inclusion criteria for this study was patients of any age presenting to Masanga hospital with a wound. We specifically included children in our ethics submission in order to capture those <15 years-old, the most typical population affected by BU in West Africa. However no patients of this age category presented, which is an interesting finding in itself.

Change(s): Discussion of this point in the discussion section, with reference to this limitation, and how future studies might address the issue (Lines 826-30)

*Reviewer #1:*

*In table 1, Participant 18 presents with a left foot ulcer which had been present for 36 months. Was this not a neuropathic ulcer related to his leprosy which he had as per table 2? How was this ruled out as a differential since patients with ‘other plausible cause of ulcer’ are listed as being excluded from the study?*

Response: Given this patient’s history of leprosy, they were assessed as to whether they had active leprosy, and this was excluded. The patient was previously treated for their leprosy, when active, with Dapsone/leprosy treatment as per local hospital protocol, and their wound did not demonstrate appearances typical of neuropathic ulcer. For these reasons the wound was not suspected to be caused by leprosy, which would have excluded them from our study as per the exclusion criteria (Lines 207-215). We decided to include this patient, on the logic that they could have a wound caused by BU despite previously having leprosy. However, we agree with the reviewers concern that the patient’s past medical history of leprosy should be taken into account, and this patient does have a higher likelihood of neuropathic ulcer in light of their previous leprosy. For this reason, we have carefully considered this dilemma and made the difficult decision based on these comments to exclude this patient (numbered participant 18 in the original manuscript), in order to avoid any potential confusion/confounding.

Change(s):

- Removal of participant (numbered participant 18 in the original manuscript) from Table 1 & 2 (Page 16 & 17), renumbering of subsequent patient from 18 to 21 (these are arbitrary numbers given to identify patients in the manuscript, which are mapped to the study IDs given in the study database)
- Update of all references to 22/twenty-two participants to reflect there being 21 participants once this participant is removed (Lines 56, 95, 335, 339, 427, 788)
- Updates of the findings/demographics/QoL/swab number recalculated after exclusion of the data from the removed participant, with 21 participants in total dataset (Lines 56-63, 339-62, 367-71, 381, 396-403, 409-10, 847-48, 847)
- Amendment of Figure 2, as B1 was the image of a histological section from the participant who has now been removed (Lines 762-79), other images updated to reflect current participant numbering.
- Explanation of this participant being excluded from the study (lines 375-79)

*Reviewer #1:*

*Technique for collecting wound swabs for BU- why swab centre of the wound?*

Response: Apologies for this typo, the swabs were taken from the undermined edges, not the centre of the wounds.

Change(s): ‘...swab specimens were taken from the lesion by passing the swab around the undermined edge rather than the centre of the lesion, to ensure that the areas with the highest load of bacteria were sampled.’ (Lines 252-55)

*Reviewer #1:*

*And why did the authors feel they will have to reconfirm (line 227) if qPCR, DSE or histopathology was positive for BU? The rationale for wanting this reconfirmation is unclear.*

Response: We agree that re-confirmation is the wrong word. For all patients we performed the three standard diagnostic methods: Histopathology from tissue, PCR and direct detection of AFB from swab samples. In case of positivity additional tests: thin layer chromatography (TLC), enzyme-linked immunosorbent assays (ELISA), and culture swabs would have been performed.

Change(s): Rewording to remove the use of the word ‘reconfirm’ to make the explanation clearer (Lines 284-99, 757-60)

*Reviewer #1:*

*Delete line 267-269 as it is of no added importance to the current manuscript.*

Response: Agree with this comment

Change(s): These lines have been deleted (was positioned at Line 336 in revised manuscript)

*Reviewer #1:*

*Was disability assessed in these patients with chronic wounds? What were the findings of the disability assessment?*

Response: Disability was assessed within the quality-of-life questionnaire, which includes questions regarding limit to mobility, stair-climbing, effect on activities of daily living, see lines 235-48. There was no separate assessment of disability.

*Reviewer #1:*

*Was this a retrospective study? It seems to me a prospective study will have obtained necessary contact details of patients for follow up and communication. How did the authors intend to disseminate test results to patients if they returned positive and therefore needed appropriate antibiotic therapy for BU?* 

Response: This was a prospective study. The contact details of each patient were collected for follow up, but attempts to contact the patient once they had left the hospital was often futile.

Change(s): Updated manuscript with reference to contact detail collection and attempts to follow up patients (Lines 231-32, 383-93, 906-25)

*Reviewer #1:*

*What was the time interval between sample collection and when results were available from the European laboratory?*

Response: The results were available five months after the samples were collected (this included transportation time, analysis etc)

Change(s): Inclusion of this information in discussion (Lines 788-793)

*Reviewer #1:*

*Did the participants receive any treatment for their wounds? How were the chronic ulcers managed? What wound care services were provided to participants? This has not been described except a brief mention in line 301 that ten lesions required surgical intervention during the study period.*

Response: Yes, the participants received regular treatment for their wounds, as per local protocol. For the majority this included regular wound dressing, and in some cases surgical intervention such as debridement.

Change(s): Inclusion of information regarding wound treatment in the methods section (Lines 191-94) and information on surgical interventions (Lines 474-78).

Inclusion of information regarding time between sample acquisition and analysis (Lines 788-793)

*Reviewer #1:*

*All 22 patients included in the study tested negative for BU on all modalities. Is this not an indication the awareness and expertise of the involved staff in recognising BU lesions is low? Some training may have been needed to improve the clinical recognition of BU especially in a setting as described where diagnostic facilities are limited. The authors should indicate practically how this can be achieved*

Response: Training was given to staff at the start of the study regarding clinical recognition, epidemiology, treatment of BU.

Change(s): Inclusion of information about the training provided and who attended (Lines 217-221)

*Reviewer #1:*

*What were the actual histopathology findings? What were they suggestive of?*

Response: The histopathology analyses of the biopsy tissue specimens revealed no AFBs in ZN-Methylene blue staining, but did show heavy infections with a broad range of other microorganisms (Lines 856-60))=. Histology images are included in Figure 2 (see Figure 2 and legend Lines 762-779)

*Reviewer #1:*

*The authors indicate a high rate of loss to follow up. Reading the methods section however, it is unclear if there was any real attempt or intent to follow up participants and what the intended purpose of any follow up was.*

Response: The contact details of each patient was collected for follow up, but attempts to contact the patient once they had left the hospital was often futile.

Change(s): Updated manuscript with reference to contact detail collection and attempts to follow up patients (Lines 231-32, 383-93, 906-25)

*Reviewer #1:
Line 292-293: Repetition. Delete ‘limited to a single leg’*

Change(s): Deleted (now Line 393)

*Reviewer #1:*
Line 301: ‘…ten had lesions requiring surgical intervention during the study period’. What was the reason(s) for the surgical intervention and what surgical procedures were performed?

Response: The indication for these surgical interventions were dependant on the individual cases, according to local hospital management protocols. Participant surgical history included: two had split skin grafts and debridement, six had split skin grafts only, and two had amputations.

Change(s): This information has been included in the manuscript (Lines 474-78)

*Reviewer #1:*

*Table 1: Needs to be redone. Why are ages and weights of individual participants presented as ranges?* 

Response: Our original table had the exact ages of participants, however it was felt that this could lead to an issue of confidentiality, as there are a number of very specific details (occupation, education level, weight, etc) which could make a patient easily identifiable. For this reason, we decided to instead give age ranges and weight ranges for each patient in our final manuscript. However, we are very happy to rework the table to include exact numbers, and look to the journal to give guidance on what level of details can be included in regard to patient confidentiality. In terms of most relevant, we feel exact ages are useful, and have therefore updated the manuscript to include exact ages, and kept the weights as ranges. Please let us know if you feel both of these require exact numbers, and we can rework the table to also include exact weights.

Change(s): Table 1 updated with exact ages (Page 16), several patients moved positions and renumbered (both in Table 1 and with the corresponding lines in Table 2) to present the list in age order (by specific ages rather than age ranges).

*Reviewer #1:
The authors state ‘Fifty percent of participants reported having previously undergone a surgical intervention, including split skin graft (36%), debridement (14%), and amputation (9%), although it was difficult to ascertain if this was at the same site as the presenting wound’. See Tables 1 and 2 for further details ’(line 313-316). There is no indication in the tables of which participants had prior surgical intervention. This must be included in the table 2. Again, although 50% were said to have had surgery, the total percentage of surgical interventions totals more than 50% [split skin graft (36%), debridement (14%), and amputation (9%)]. Why is this so?* *Why is it difficult to ascertain the site of surgical interventions like grafting or an amputation?*

Response:

We agree that stating these as percentages has made this is confusing, as some patients had split skin grafts and debridement. Furthermore we agree it would be useful to list this information in the tables.

Change(s):

Rewording of this section, by breaking down the surgical interventions by numbers of patients rather than percentages (Lines 474-78). Update of Table 2 with a column for surgical history (Page 17). Removal of comment regarding ascertaining the site of surgical intervention.

*Reviewer #1:*

*What was the interval between the reported surgical intervention and the time of sampling for BU confirmation? This is an important consideration that can impact results of laboratory confirmation of BU*

Response: Any delays in sample processing following biopsy was only due to firstly sample storage to allow transportation and analysis of all samples as a batch, and secondly due to the logistics and time involved in transportation. As a result, the final laboratory results were available five months following samples acquisition. This delay is unlikely to have influenced the results, however it does highlight the need for point-of-care rapid diagnostic/lateral flow tests for M. ulcerans.

Change(s): Inclusion of this information in discussion (lines 786-93) as well as reference to the temperature stability during transportation (Lines 747-49).

*Reviewer #1:*

*It is not clear what the authors seek to achieve by providing previous histories of malaria and yellow fever. Is there a relationship with the current wounds?
In Table 2, several persons are reported to have weight loss; in association with chronic ulceration, is there a suspicion of the ulcers being malignant?*

Response: The past medical history section includes all medical history that was felt to be relevant to wound development caused by a range of aetiologies. There was not necessarily a direct relationship with the current wounds, or a suspicion of the ulcers being malignant. The past medical history is included for completeness.

Change(s): Inclusion of the general and specific past medical history questions in Table 2 with a note related to two asterisk ** in column 2 which underneath lists all the specific medical problems the patient was asked if they suffered with currently or previously (Page 17) Clarification of there being no suspicion of malignancy in the results section (Line 369-79).

*Reviewer #1:*

*Again, under weight loss column, some are described as ‘incomplete’. What does that mean? 
Participant 17 has ‘incomplete’ palpable lymph nodes. Meaning?*

Response: This is due to a lack of recording of these pieces of information for this particular patient. The fact that these items were missed was only identified after the patient had been discharged and were uncontactable to return for inclusion of these data points. Nonetheless, it was decided to keep the patient in the study as they fit the inclusion criteria and had all other data points collected.

Change(s): Changed ‘Incomplete’ to ‘Not recorded’ (Table 2, Page 17). Updated the results with the above explanation (Lines 371-79).

*Reviewer #1:*

*Line 328-329: Which specific cocci and rods were found to be present in the wounds? This is important to indicate*

Response: We did not do specific staining or performed culture to determine the bacterial species present in tissue samples. Tissue samples and swabs were analysed more than 4 weeks later. Methylene blue staining detects DNA and therefore we could see that cocci and rods were present in a significant amount, and we have included photographs to demonstrate this. We agree that for future studies bacterial cultures at the site of investigation should be performed as it was done in the following paper:

Secondary bacterial infections of buruli ulcer lesions before and after chemotherapy with streptomycin and rifampicin. Dorothy Yeboah-Manu, Grace S Kpeli, Marie-Thérèse Ruf, Kobina Asan-Ampah, Kwabena Quenin-Fosu, Evelyn Owusu-Mireku, Albert Paintsil, Isaac Lamptey, Benjamin Anku, Cynthia Kwakye-Maclean, Mercy Newman, Gerd Pluschke. PLoS Negl Trop Dis. 2013 May 2;7(5):e2191.

Change(s): Added this limitation to the discussion section (Lines 856-60)

*Reviewer #1:*

*Line 371. Change to ‘a well-known risk factor…
(Line 379-380): How does this study ‘address a knowledge gap in the understanding of the aetiology of chronic lower limb ulceration in Sierra Leone’? 
Line 394-396: ‘In addition, no participants presented with nodules or plaques, which may be because these are typically painless and therefore without an impetus to seek treatment if knowledge of BU is lacking in the community’. This is not a reasonable conclusion from this study as the initial study population comprised patients presenting with only wounds/ ulcers in the hospital. Please delete*

Change(s): Changed to ‘well-known’ (Line 815)

Reworded paragraph about addressing knowledge gap and removed sentence regarding reduced presentations of nodules to this hospital (Lines 823-827).

*Reviewer #2:*

*This paper described the result of the search of Buruli ulcer in an Hospital of Sierra Leone. It resulted that none of the lesions examined were not consistent with BU. The Authors, thus, wrightly, concluded that, this negative result didn’t discard existence of BU in Sierra Leone. Indeed, the relevance of this study, given the fact that participants are not recruited from a community case search study, and the very limited number of participants, are valid only for this study site, namely the Masanga Hospital. We, then, suggest to the authors, to review the scope of the study, reformulating the title, to limit it to the hospital site*

Change(s): Titles amended as suggested (Lines 3-5); these identified limitations are conferred in the Discussion section (Lines 927-31).

We are very grateful for your consideration of our updated manuscript as per the comments from your reviewers. We hope you are satisfied with our changes, and look forward to your correspondence regarding whether our manuscript submission is successful.

Kind regards,

Helen Please and co-authors
